# Multifocal Small Bowel Angioectasias: Managed with Innovative, Nonresectional Surgical Procedure

**DOI:** 10.1055/s-0042-1744151

**Published:** 2022-08-16

**Authors:** Nalini Kanta Ghosh, Ashish Singh, Rahul Rahul, Rajneesh Kumar Singh, Amit Goel, Rajan Saxena

**Affiliations:** 1 Department of Surgical Gastroenterology, Sanjay Gandhi Postgraduate Institute of Medical Sciences, Lucknow, Uttar Pradesh, India; 2 Department of Medical Gastroenterology, Sanjay Gandhi Postgraduate Institute of Medical Sciences, Lucknow, Uttar Pradesh, India

**Keywords:** small bowel angioectasias, lower GI bleeding, transmural sutures

## Abstract

Gastrointestinal (GI) angioectasias/angiodysplasias are the most frequent vascular lesions of GI tract, responsible for ∼5 to 6% of GI bleedings. It commonly involves the small bowel, making it difficult to diagnose and manage endoscopically. Though medical management has been used to prevent bleeding, it has only a limited role in acute severe hemorrhage. In such cases, surgical resection remains the only practical option. However, multiple lesions pose a unique challenge, as resection may not be advisable for long length of bowel involvement. Here, we report a case of recurrent GI bleeding due to multifocal small bowel angioectasias who was managed by a novel technique of full-thickness transmural sutures under intraoperative enteroscopic guidance. At 6 months follow-up, no new bleeding episodes were observed.

Gastrointestinal angioectasias (GIAs)/angiodysplasias are the most frequent vascular lesions of GI tract and are responsible for ∼5 to 6% of GI bleedings. 1 It is most common in the proximal small bowel. 2 Diagnosis is usually made with endoscopy, computed tomography (CT), or magnetic resonance imaging (MRI) scans. Small bowel being the most common site, it is difficult to diagnose and manage endoscopically. If lesions are accessible and amenable to endoscopic treatment, it is preferred over the more invasive surgical management. In lesions that are refractory or inaccessible to endoscopic management, surgery is the only recourse left. Surgical management usually involves resection of a variable length of lesion bearing bowel. Multifocal lesions pose a challenge to extensive surgical resection, as it may not be feasible to resect all the lesions. We present a case of multifocal small bowel angioectasias distributed throughout the small bowel, who presented with recurrent episodes of lower GI bleeding and was managed surgically with multiple full-thickness sutures under intraoperative enteroscopic guidance.

## Case Summary

**Fig. 1:**
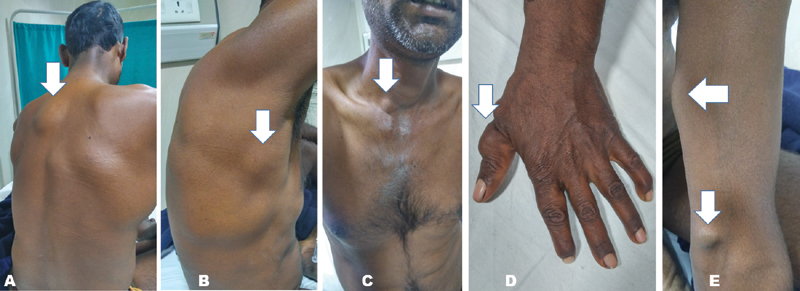
Multiple soft tissue swellings ( **A** —back, **B** —right chest wall, **C** —supraclavicular area, **D** —left thumb, **E** —left cubital fossa). Arrows showing the subcutaneous lesions.

**Fig. 2:**
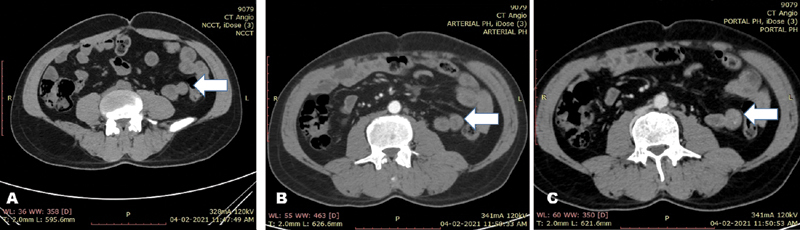
Vascular malformations (lesions filling in portal venous phase) in triple phase computed tomography scan (small bowel [ **A** —noncontrast, **B** —arterial, **C** —portal venous phase]). Arrow showing the bowel loop with vascular malformation at different phases of CT scan.

**Fig. 3:**
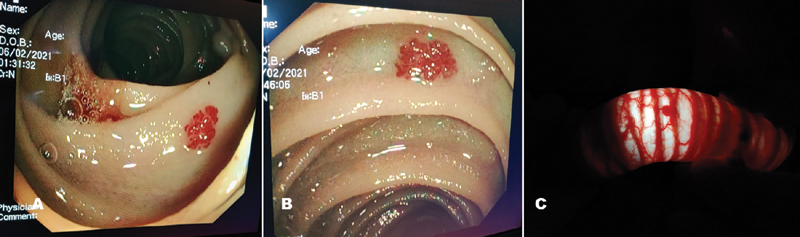
Intraoperative pictures showing angioectasias ( **A** , **B** ). ( **C)** Transillumination showing angioectasia with feeding vessels.

**Fig. 4:**
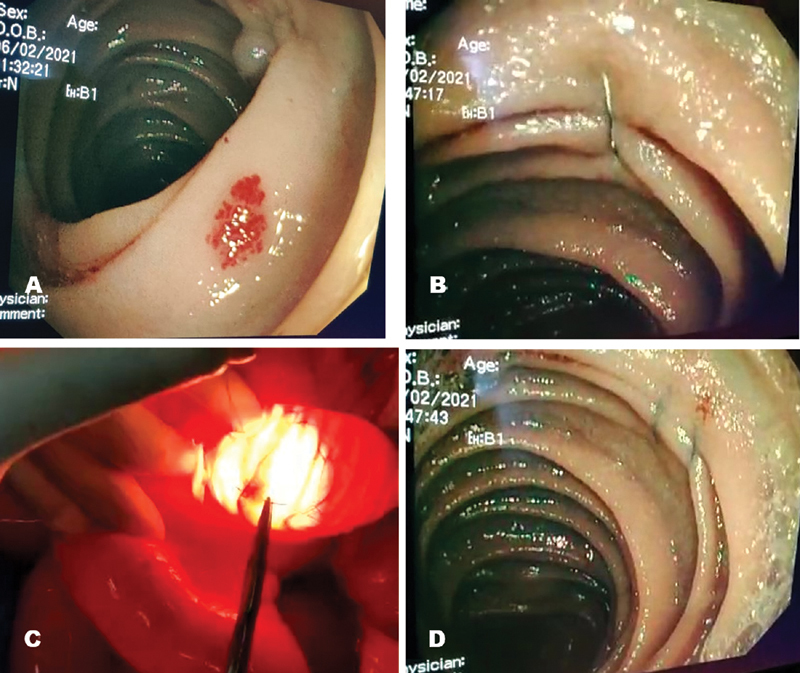
Suture ligation of angioectasia. (A) Enteroscopic view of angioectasia, (B) Suture being taken under enteroscopic guidance, (C) Transillumination showing same angioectasia, being suture ligated from outside (full thickness), (D) After suture ligation enteroscopic view.

A 37-year-old male presented with history of intermittent episodes of melena for the past 9 years. First episode was at the age of 28 years, when he had massive lower GI bleeding requiring 10 units of packed red blood cell transfusion and was managed with surgical resection of small bowel segment at another hospital. Again, at the age of 32 and 35, he had melena, which stopped spontaneously with conservative treatment and no blood transfusion was required. In the present episode, the patient presented with 10 days history of melena with 8 g/dL drop in hemoglobin (from 12 g/dL to 4 g/dL). He had no pain abdomen, vomiting, fever or any history of trauma. Additionally, the patient had also noticed multiple painless subcutaneous swellings all over his body (larger ones being over the neck and chest), since childhood. Examination revealed moderately built and nourished male (body mass index: 21.4 kg/m ^2^ ), who was pale looking and tachycardic (heart rate—110/minute) with multiple painless soft tissue swellings all over his body ( Fig. 1 ). These were compressible and suggestive of vascular malformations, although no bruit could be appreciated on auscultation. Abdominal examination was unremarkable. Digital rectal examination confirmed melena. Liver function, renal function, and coagulation profile tests were normal. He was evaluated with upper GI endoscopy, which showed normal findings till second part of duodenum. Colonic mucosa was obscured with melanic stools on colonoscopy. CT angiogram revealed mucosal vascular ectasias in proximal small bowel ( Fig. 2 ). In view of massive lower GI bleeding, he was planned for exploratory laparotomy and intraoperative enteroscopy. Laparotomy revealed that small and large bowel were filled with luminal blood. Previous anastomosis site was identified two feet from duodenojejunal junction. Intraoperative enteroscopy was done using adult gastroscope through a mid-small bowel enterotomy, to examine the entire small bowel. It revealed multiple angioectasias (3–10 mm patchy lesions without protrusion) more than 20 in number (type 1b according to Yano Yamamoto classification 3 ) distributed throughout the small bowel with few showing active bleeding ( Fig. 3 ). Resection of such a long length of bowel was not considered advisable. Hence, under guidance of intraoperative enteroscopy full-thickness sutures (polypropylene 3–0, figure of eight or running sutures for longer lesions) were taken from serosal aspect over both bleeding and nonbleeding angioectasia lesions ( Fig. 4 ). The bowel enterotomy was then primarily closed. Postoperative course was uneventful with no further bleeding. On regular follow-up of more than 6 months, he is doing well with no further bleeding episode.

## Discussion

GIAs are vascular malformations composed of dilated and tortuous arterial or venous capillaries. They are usually smaller than 5 mm in diameter and located in the mucosal and submucosal layers of the GI tract. These are mostly asymptomatic and the incidence on endoscopic studies varies from 1 to 5%. 4 5 To differentiate between venous/capillary lesions (angioectasias), arterial lesions and arteriovenous lesions on endoscopic view Yano Yamamoto classification categorizes these lesions in to venous/capillary lesions (angioectasias), arterial lesions or arteriovenous lesions on the basis of endoscopy. According to which, lesions in our patient comes into the category of angioectasias (type 1b). GIA may be part of hereditary syndrome (such as hereditary hemorrhagic telangiectasia), or may be acquired (such as gastric antral vascular ectasia, radiation-induced vascular ectasia, Dieulafoy's lesion, and angioectasias). 6 Though GIAs are more commonly found in elderly patients (>60 years old), our patient was a young (37 years) male with first episode of GI bleeding at the age of 28 years. He had multifocal angioectasias distributed throughout the small bowel along with multiple subcutaneous vascular lesions (multiple soft tissue compressible swellings all over the body) that suggested a possible genetic association. However, workup and family history did not provide any lead to genetic syndrome. GIAs in association with cutaneous and subcutaneous vascular malformations have been described in blue rubber bleb nevus syndrome, 7 but in these case cutaneous lesions are predominant presentation that are usually small blue-colored lesions and they present in early life with chronic anemia requiring lifelong iron therapy and blood transfusions. In contrast, our patient had first presentation at the age of 28 years with recurrent massive GI bleeding with no cutaneous lesions but with large subcutaneous lesions in form of swellings, although the multiplicity of the GI lesions is a common finding. A definite association between bleeding GIAs and aortic stenosis (Heyde's disease) and other valvular heart disease has been clearly described. 8 9 Various other risk factors have been identified such as chronic renal failure, chronic liver disease, and Von Willebrand disease. 10 11 Preoperative workup of our patient revealed a normal echocardiography and other biochemical parameters. In literature, the diagnosis is usually made with small bowel enteroscopy (single balloon, double balloon, spiral or video capsule enteroscopy). Though axial imagings such as contrast-enhanced CT/MRI are known to have a low sensitivity for GIAs, 12 in our patient CT angiography provided specific finding of enhancing mucosal lesions pointing to the preoperative diagnosis of GIAs as the cause of bleeding. Management of bleeding angioectasias is a challenge and there is no formal guideline available. 13 14 Treatment may be divided into hemostatic, rescue therapy (when all treatment fails) and prophylactic. First-line hemostatic treatment is endoscopic (mechanical/thermal-contact [monopolar/bipolar) and noncontact APC (Argon plasma coagulation)/laser]. Among these, APC is the preferred method. 15 Highly selective angioembolization has also been used; it is at the risk of a high incidence of complications (perforation/gangrene). Rescue therapy (surgical management) is required when all other forms of managements fail or in life-threatening situations due to massive bleed, as was in our case. However, morbidity and mortality of a surgical intervention remain high as these patients are elderly and often have major comorbidities. 16 Although resection of the involved bowel segment is the only described surgical option, in presence of multiple lesions an extensive bowel resection may not be advisable. Hence, in our case, instead of an extensive bowel resection, we chose to perform transmural ligation of all the lesions (bleeding as well as nonbleeding) under enteroscopic guidance. To the best of our knowledge, this surgical technique has not been reported earlier and provided a novel method to secure multiple bleeding angioectasias in the small bowel without any added morbidity of extensive bowel resection.

In some patients, recurrent bleeding has been observed even after the best possible invasive treatment. This is probably because of the multiplicity of the lesions and possible reappearance of new lesions. In such cases, pharmacotherapy may be useful for secondary prophylaxis of bleeding GIAs. Various pharmacological agents have been proposed and tried like hormonal drugs (mixture of estrogen and progesterone), somatostatin analogue (octreotide, lanreotide), or thalidomide. 17 18 19 20 Apart from thalidomide, none of the agents have been found effective in studies. 19 20 21 In our patient, we controlled all the visible lesions surgically and he has not had any recurrence of bleeding till last follow-up; hence, we have not given thalidomide therapy as secondary prophylaxis but reserved thalidomide for use in case of recurrent bleeding in this patient.

## Conclusion

Bleeding of small bowel angioectasias poses a major challenge in view of location and multiplicity, and is difficult to manage with endoscopic intervention. Although resection appears to be a definite management option in cases where surgery is required, in presence of diffusely distributed lesions throughout the small bowel, it may not be an advisable approach. Here, we report such a case of multifocal small bowel angioectasias, who was managed surgically with multiple full-thickness sutures under intraoperative enteroscopic guidance, with good outcome. This case highlights the effectiveness of this innovative surgical procedure without any added morbidity of extensive bowel resection.
